# A Novel Laser and Video-Based Displacement Transducer to Monitor Bridge Deflections

**DOI:** 10.3390/s18040970

**Published:** 2018-03-25

**Authors:** Miguel A. Vicente, Dorys C. Gonzalez, Jesus Minguez, Thomas Schumacher

**Affiliations:** 1 Department of Civil Engineering, University of Burgos, c/Villadiego, s/n. 09001 Burgos, Spain; dgonzalez@ubu.es or dorys.gonzalezcabrera@slu.edu (D.C.G.); jminguez@ubu.es (J.M.); 2 Department of Civil Engineering, Parks College of Engineering, Aviation and Technology, Saint Louis University, 3540 Lindell Blvd, Saint Louis, MO 63103, USA; 3 Department of Civil and Environmental Engineering, Portland State University, 1930 SW 4th Ave, Portland, OR 97201, USA; thomas.schumacher@pdx.edu

**Keywords:** displacement sensor, laser beam, digital video camera, static deflection, long-term monitoring, bridge

## Abstract

The measurement of static vertical deflections on bridges continues to be a first-level technological challenge. These data are of great interest, especially for the case of long-term bridge monitoring; in fact, they are perhaps more valuable than any other measurable parameter. This is because material degradation processes and changes of the mechanical properties of the structure due to aging (for example creep and shrinkage in concrete bridges) have a direct impact on the exhibited static vertical deflections. This paper introduces and evaluates an approach to monitor displacements and rotations of structures using a novel laser and video-based displacement transducer (LVBDT). The proposed system combines the use of laser beams, LED lights, and a digital video camera, and was especially designed to capture static and slow-varying displacements. Contrary to other video-based approaches, the camera is located on the bridge, hence allowing to capture displacements at one location. Subsequently, the sensing approach and the procedure to estimate displacements and the rotations are described. Additionally, laboratory and in-service field testing carried out to validate the system are presented and discussed. The results demonstrate that the proposed sensing approach is robust, accurate, and reliable, and also inexpensive, which are essential for field implementation.

## 1. Introduction

In the last 60 years there has been a spectacular increase in bridge heritage issues. While these infrastructures have provided significant benefits to society, an increasing amount of resources are needed for their maintenance in order to ensure an adequate level safety and comfort. Within the maintenance tasks, visual inspection is an essential activity, ultimately enabling efficient resource use. Challenges with this activity have been associated with the need for a significant use of human resources combined with low efficiency. Additionally, in many cases inspection results show a notable level of subjectivity, that is, in most cases the bridge inspection results depend on the skill and experience of the technicians who performed the inspection.

On the other hand, there has been enormous development of sensor technology, increasing their quality and, overall, dramatically reducing their cost, which has allowed their use for bridge monitoring applications. Bridge monitoring becomes a useful tool, alongside an inspection with its conventional procedures, to quantify the actual state of the bridge and its evolution over time. Using the monitoring results it is possible to implement better bridge preventive maintenance plans, which can be also specific for each structure. Among the many parameters that can be monitored on a bridge, vertical deflection is perhaps the most useful one, as it can be directly related to the in-service behavior of a bridge. First, all international standards specify the maximum allowable deflection of a bridge under service loads to ensure its functionality [1,2]. In addition, aging processes, mechanical degradation, corrosion, and deferred phenomena (creep and shrinkage in case of concrete bridges or loss of prestressing in case of prestressed/post-tensioned bridges) directly impact vertical deflection. However, long-term monitoring of the vertical deflection of bridges under service continues to be a first-level technological challenge, especially in case of long and/or tall bridges and/or if they are in difficult-to-access places. Finally, bridges are exposed to harsh environmental conditions and exposed to sever weather. The ideal measurement system is thus:

- accurate, with the ability to measure small displacement changes;
- robust, to withstand varying temperature and humidity and operate under harsh conditions;
- reliable, producing accurate and repeatable measurements; and
- inexpensive, due to the large number of bridges to be monitored.

Several technical solutions exist on the market such as: displacement transformers (e.g., LVDT), laser distance meters, acoustic and electromagnetic interferometers, total station theodolites, global positioning system (GPS)-based systems, geophones, accelerometers, etc. However, none of them are able to fulfill all the requirements previously described.

In recent years, a novel set of solutions has emerged: video-based sensors. This promising and quickly evolving technology has been driven by the continuous improving of the features of digital video cameras resulting in rapidly decreasing costs. Today it is possible to get a commercially-available 4K video camera at accessible prices. In addition, the development and wide availability of digital image processing (DIP) software, initially developed for applications very different from bridge monitoring, allows one to simplify post-processing tasks to extract the required information. While this is a promising technology, it is not a technology mature enough to be widely implemented for bridge monitoring purposes. Several challenges still exist with this technology before it can be implemented in the field. The first one is concerning resolution and accuracy, which have to be further improved in order to reach a level of, for instance, an LVDT. The second one is the simplification of post-processing tasks. A video record is usually a large file and contains a significant amount of excessive information with only a small portion of it being useful. In order to be able to monitor a structure in real time by analyzing a video record, the ability to post-process many frames per second (usually 60 fps with a size of 3840 × 2160 pixels) is critical.

In most of the research published up to date, the video camera is placed away from the bridge on a fixed location. Typically, the bridge is equipped with one or several targets. The movement of these targets is obtained through the comparison of individual video frames [3,4,5,6,7,8,9]. While the targets can be tracked, any fixed reference cannot. This solution has some relevant limitations. The first one is related to the accuracy of the solution. In real bridges it is expected that the video camera will be placed far from the bridge and, in consequence, the pixel size (which is primary related to the accuracy of this solution), is thus large. For the case of very large bridges where the expected deflections are large, i.e., on the order of tens or hundreds of millimeters, this represents a feasible solution [10,11]. For the case of stiffer bridges with expected deflections in the millimeter and sub-millimeter range, however, it may not work. A natural way to reduce the pixel size is the addition of large (and expensive) lenses to reduce the vision-field of the camera and, in consequence, the pixel size. The second one is that it is not possible to monitor during night time. A fundamental assumption for this family of solutions is that the video camera remains fixed over time, which may not be the case. There is another approach, alternative to the previous one. In this case, the video camera is located on the structure moving jointly with the target and the fixed reference is visible in the field of view of the video camera [12]. From the video camera point of view, the fixed reference moves showing an opposite movement of the target.

A relevant amount of the research published uses video-based sensors to measure the dynamic response of structures, that is, natural vibration frequency and damping ratio, among others. Also, the measure is carried out during short periods of time on the order of seconds or minutes. The results have been compared to accelerometers and/or LVDT [13,14,15,16,17,18,19,20]. The measurement of the short-term dynamic response of a bridge is technologically less demanding than the one required to capture long-term changes. In addition, this is a less useful solution because accelerometers fulfill all the requirements to obtain such measurements: they are robust, accurate, reliable, and inexpensive.

Recently, some interesting work has been carried out to measure inclination, alone or in combination with displacements, using lasers and videos [21,22,23]. The results were compared to conventional inclinometers. However, this approach has the same limitation as the previous one: inclinometers are already a robust, accurate, reliable and inexpensive solution.

However, this body of work is a useful starting point for the development of a video-based technology capable of capturing static and slow-varying displacements over long periods of time. Only a few number of researchers have documented the monitoring of static vertical deflections over a period of days or weeks [24].

It is worth highlighting in more detail the research performed by Zhao et al. [25,26]. In their work, a laser device was placed in the fixed position instead of the video camera, which was located on the structure. The device emits a laser beam, which projects a dot on a projection plate within the field of vision of the video camera. The video camera moves jointly with this projection plate and both of them move jointly with the target. When the target moves, the video camera records a movement of the laser dot, which is opposite the movement of the target. One of the main advantages of this solution is that low technical requirements are needed for the video camera (no optical zoom, no high resolution, etc.) because the distance between the projection plate and the video camera is small and hence also its field of vision. In consequence, the size of a pixel is small. The researchers demonstrated that it is possible to perform monitoring using a conventional smartphone. This approach, however, still has some weak points. First, the projection plate is opaque. In consequence, both the laser device and the video camera are placed on the same side of the projection plate, which causes the laser beam to have an oblique impact angle on the projection plate. This must be considered when computing the movement of the target. Second, it is required that the target, the projection plate, and the video camera move jointly; otherwise it is not possible to calculate the movement of the target. The reason for this is that inside the field of view only the fixed reference (laser dot) is included.

To the best of the authors’ knowledge, none of the solutions developed to date are capable of tracking a fixed and a moving reference within the field of vision of the video camera simultaneously. This paper introduces a novel sensing approach to monitor displacements and rotations on bridges and structures by combining a laser beam, a video camera, and LED lights. This solution substantially improves the approach proposed by Zhao et al. [25,26], eliminating the limitations explained earlier. Additionally, the proposed solution can used any time of the day, day or night, because both the fixed and the movable reference are light-emitting. Subsequently, the proposed laser and video-based transducer (LVBDT) is described in detail. A laboratory experiment and an in-service field test to evaluate the solution’s feasibility are presented and the results discussed.

## 2. Proposed Sensing Approach

### 2.1. Equipment and Components

**Figure 1:**
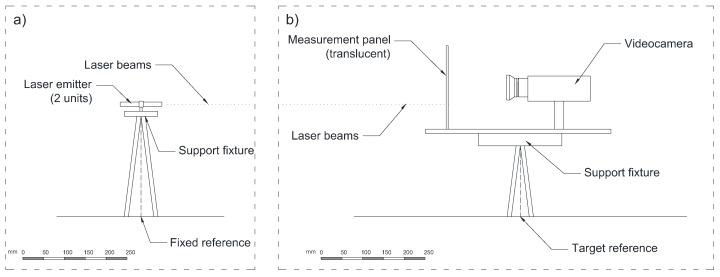
Illustration of laser and video-based displacement transducer (LVBDT): (**a**) Fixed part, located on fixed reference (e.g., non-moving location away from bridge); (**b**) Movable part, located on target reference (e.g., bridge girder at mid-span).

The proposed laser and video-based displacement transducer (LVBDT) is composed of two main components: the fixed part and the movable part, as illustrated in Figure 1.

The fixed part is placed at a non-moving location and to remain fixed at all times. It is composed by a support fixture holding two laser emitters. The laser emitters used in this research are green dot lasers with a wavelength of 532 nm, output power 5 mW, class IIIA and a beam divergence of 1 mrad.

The movable part is placed on the structure and moves jointly with it. It is composed of a video camera and a measurement panel placed on a support fixture. The video camera used in this research was a Lumix DMC-G80 (Panasonic, Osaka, Japan) with 16 MP and image stabilization, type 5-axis/5-stop, Dual IS, with a recording speed of 60 fps and an optical zoom of 12–60 mm f/3.5–5.6. The video camera was not connected to a computer, although this is an option that could be implemented.

**Figure 2:**
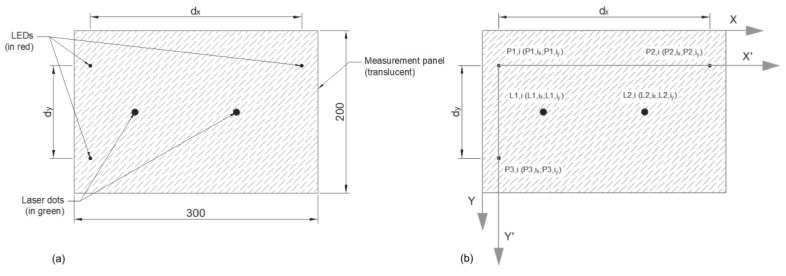
Measurement panel: (**a**) description and dimensions and (**b**) coordinate axes.

The measurement panel is made of transparent methacrylate with length × height = 300 mm × 200 mm and a thickness of 5 mm. On the backside, a white paper sheet was added to make the measurement panel translucent. Additionally, the panel is equipped with three red LED lights (Figure 2a).

The two laser beams project dots on the measurement panel on the opposite side of the white sheet. Inside the field of vision of the video camera there are five references, which are, the three red LED lights and the two green laser dots (Figure 2a). The three LED lights define a Cartesian coordinate system (Figure 2b). The dimensions *d_x_* and *d_y_* corresponding to the distances between the LED lights are constant and known. The measurement panel should be placed orthogonally to the video camera. Additionally, the zoom of the video camera should be in its most open position in order to minimize the fish eye effect. The field of vision of the video camera should be completely inside the measurement panel and it should include the five previously mentioned references. When movement of the target occurs and, in consequence, the same movement of the measurement panel, the fixed references on it (laser dots) remain fixed, while the movable references (LED lights) move. However, the record of the video camera will show, in the most general case, that both fixed and movable references move (in fact, this happens when the video camera moves respectively to the measurement panel). In the desired case the video camera and the measurement panel move jointly and the record of the video camera will show that the fixed references move while the movable references do not move.

An ideal measurement system must be robust; otherwise it cannot be used on real structures and for long-term monitoring. The robustness of our system depends, among others, on the laser device. The market provides laser pointers able to withstand environmental conditions and with high pointer stability. If necessary, the laser device can be protected using a cover with a transparent window.

The system can monitor displacements and rotations in plane with the measurement panel plane. Movements and rotations out of the measurement panel plane cannot be captured.

The recommended range for the laser emitters used in this study is below 30 m in order to obtain a laser dot diameter below 30 mm. When the distance between the fixed and the movable reference is larger, low divergence laser emitters should be used, with a divergence of 0.3 mrad or less. Additionally, a greater measurement panel should be used, in order to ensure that the laser dots remain inside the measurement panel during the entire monitoring process.

### 2.2. Computational Approach

Next, the step by step numerical procedure to analyze the video data is described in detail.

#### 2.2.1. Video-to-Frames Extraction

First, the video camera records the five references previously explained, i.e., the three LED lights and the two laser dots and many other useless information. The video is recorded using an appropriate frame rate, e.g., at a speed of 60 fps for slowly-varying displacements, or at high speed using special video cameras when vibrations are to be captured. The possibility to record time-lapse videos is interesting, especially for long-term monitoring and slow movement. As explained before, the field of vision of the video camera is inside the measurement panel. Therefore, the videos include three distinct colors: white, red, and green, which simplifies the post-processing of the data. The recording file of the video camera, in AVI, MPEG, MOV, WMV, or similar format is, first, decomposed into a sequence of images or frames. In this case the MATLAB software (Mathworks, Inc., Natick, MA, USA) was used. Once all frame files are obtained, the post-processing procedure is applied to each individual image. When the video is recorded in UHD, each frame has 3840 × 2160 pixels. With this current setup, the field of vision is governed by the size of the measurement panel (300 × 200 mm), which results in a pixel size of approximately 0.08 mm.

#### 2.2.2. Homography Transform

Although it is expected that the video camera remains perpendicular to the measurement panel during all the measurement time, a misalignment may occur due to unexpected actions. In consequence, it is necessary to consider this event and to include a first step in the computational procedure to correct it.

**Figure 3:**
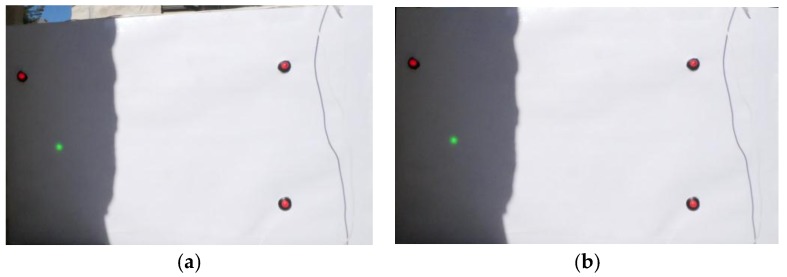
Sample frame before (**a**) and after (**b**) application of homography transform.

Neither displacement nor rotation of the frame are required, since both the fixed and the movable references are inside the frame. Additionally, it is not necessary to re-scale the frame, because the physical distance between the LED lights is known (this is intrinsic information of the sensor) and the ratio between the distance between LED lights in pixels and in mm is obtained in each frame, as explained later. In consequence, the only correction to be applied is the homography transform [27,28,29,30,31]. This transform recovers the orthogonality between the video camera and the measurement panel. Figure 3 shows a sample frame before and after application of the homography transform.

#### 2.2.3. Threshold Color Filtering

The second step is to identify, in each frame, the pixels belonging to the five references previously explained, i.e., the three LED lights and the two laser dots. To achieve that, it is necessary to filter the frames, in order to delete any unnecessary information and, thereby, reduce the size of the frames.

One of the main advantages of the solution shown is that the references are light-emitters, which implies that it is able to work under variable and changing natural lighting conditions, including night. To validate this assumption, the performance of the LVBDT was evaluated under three significantly different scenarios: during a sunny day, at night, and with the presence of a shadow on the measurement panel.

**Figure 4:**
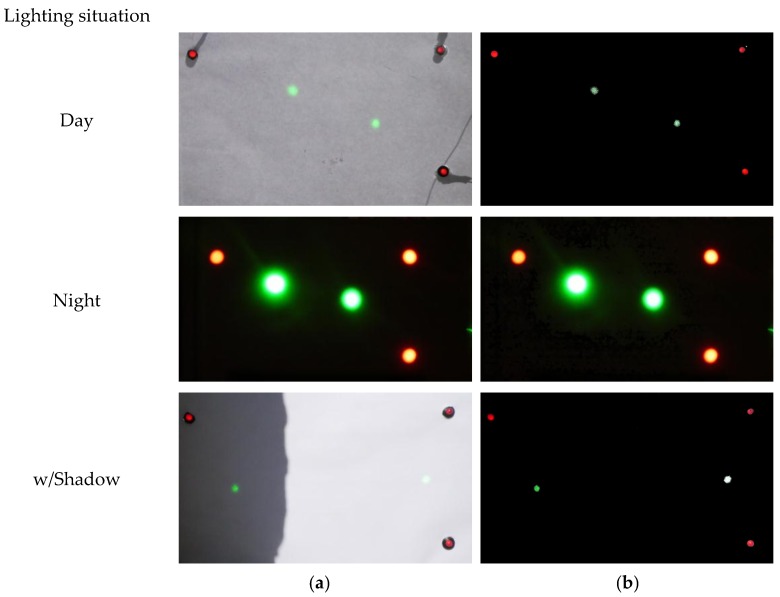
Sample frame before (**a**) and after (**b**) application of threshold color filtering for different lighting situations.

All frames were subjected to a threshold color procedure. In this case, the thresholding method proposed by Huang and Wang [32] was used. The threshold color selected was black and the color space used was hue-saturation-brightness (HSB). The hue range was 0–255, the saturation range was 0–140 and the brightness range was 0–243. Figure 4 shows a comparison between unprocessed and processed frames for the three lighting situations.

As can be seen in Figure 4, the results obtained in this processing step are satisfactory. In addition, the file size of each frame is approximately 10% of the original file size, which is beneficial from a computational point of view.

#### 2.2.4. Pixel Identification and Grouping

Next, each frame is transformed into an *N*-row, 5-column matrix where *N* is the total number of frames in the video. The first and second columns include the *X* and *Y* coordinates of a pixel, respectively, and columns three to five include the RGB color values. Each row is associated with one specific pixel in a frame. The matrix is identified using the time of the frame, which is (*n* − 1)/*t*, where *n* is the number of the frame and *t* is the time interval between frames (or inverse of the frame rate). Next, the pixels belonging to the five different references are identified and grouped. To do so, color range and proximity criteria are used. A set of color-number thresholds are defined. One color-number threshold value is defined for the laser dots and the other one for the LED lights. These thresholds are the same for all frames. The result are five *N_ref_*-row, 2-column matrices, where *N_ref_* is the number of pixels belonging to each reference point, i.e., LED or laser dot. The rest of the pixels are deleted.

#### 2.2.5. Determination of the Center of Gravity

The next step is to obtain the coordinates of the center of gravity of each reference. Procedures to achieve that are available and described in the literature [33,34,35,36,37,38]. It should be noted that sub-pixel resolution can be reached using these procedures, which implies, in this particular case, a resolution better than 0.08 mm. In this work, the weighted center or gravity has been defined according to the following expressions [38]:

(1) $${\hat{X}}_{CoG}=\frac{\sum x_{i}\cdot I_{i}}{\sum I_{i}}$$

(2) $${\hat{Y}}_{CoG}=\frac{\sum y_{i}\cdot I_{i}}{\sum I_{i}}$$

where ${\hat{X}}_{CoG}$ and ${\hat{Y}}_{CoG}$ are the coordinates of the center of gravity of the reference, $x_{i}$ and $y_{i}$ are the center of gravity of each pixel ($i≔1\;to\;N_{ref})$, and $I_{i}$ is the intensity of the pixel. This value is obtained from the ordinate axis of the red or green histograms (depending of the reference) belonging to the red or green value of the pixel.

At this point, all the information provided by a frame is concentrated in a 5 × 2 matrix and associated with the time of the frame. For the *i*th-frame, each row includes the *X* and *Y* coordinate values of the center of gravity of each of the five references, which are the LED lights *P*1,*i*, *P*2,*i*, and *P*3,*i* and the laser dots *L*1,*i* and *L*2,*i*:

(3) $$\left[\begin{array}{cc} \begin{array}{c} P1,i^{\left(0\right)}{}_{x}\\ P2,i^{\left(0\right)}{}_{x} \end{array} & \begin{array}{c} P1,i^{\left(0\right)}{}_{y}\\ P2,i^{\left(0\right)}{}_{y} \end{array}\\ \begin{array}{c} P3,i^{\left(0\right)}{}_{x}\\ L1,i^{\left(0\right)}{}_{x}\\ L2,i^{\left(0\right)}{}_{x} \end{array} & \begin{array}{c} P3,i^{\left(0\right)}{}_{y}\\ L1,i^{\left(0\right)}{}_{y}\\ L2,i^{\left(0\right)}{}_{y} \end{array} \end{array}\right]$$

Next, a transformation is performed on the matrix shown in Equation (3), including translation and rotation, from the *X*-*Y* coordinate system of the frame to the *X*’-*Y*’ coordinate system of the reference points *P*1,*i*, *P*2,*i*, and *P*3,*i* (Figure 2b). The resulting matrix is as follows:

(4) $$\left[\begin{array}{cc} \begin{array}{c} 0\\ P2,i^{\left(1\right)}{}_{x} \end{array} & \begin{array}{c} 0\\ 0 \end{array}\\ \begin{array}{c} 0\\ L1,i^{\left(1\right)}{}_{x}\\ L2,i^{\left(1\right)}{}_{x} \end{array} & \begin{array}{c} P3,i^{\left(1\right)}{}_{y}\\ L1,i^{\left(1\right)}{}_{y}\\ L2,i^{\left(1\right)}{}_{y} \end{array} \end{array}\right]$$

Next, conversion factors are applied to the matrix shown in Equation (4), in order to convert pixel coordinates to physical coordinates:

(5) $$P2,i^{\left(1\right)}{}_{x}=d_{x}$$

(6) $$P3,i^{\left(1\right)}{}_{y}=d_{y}$$

**Figure 5:**
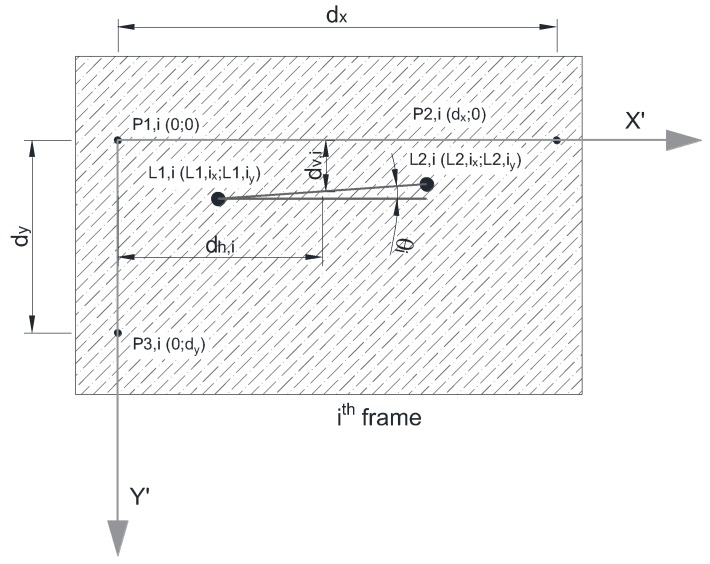
Relative positions.

The matrix is now defined as follows (see Figure 5):

(7) $$\left[\begin{array}{cc} \begin{array}{c} 0\\ d_{x} \end{array} & \begin{array}{c} 0\\ 0 \end{array}\\ \begin{array}{c} 0\\ L1,i^{\left(1\right)}{}_{x}\cdot \frac{d_{x}}{P2,i^{\left(1\right)}{}_{x}}\\ L2,i^{\left(1\right)}{}_{x}\cdot \frac{d_{x}}{P2,i^{\left(1\right)}{}_{x}} \end{array} & \begin{array}{c} d_{y}\\ L1,i^{\left(1\right)}{}_{y}\cdot \frac{d_{y}}{P3,i^{\left(1\right)}{}_{y}}\\ L2,i^{\left(1\right)}{}_{y}\cdot \frac{d_{y}}{P3,i^{\left(1\right)}{}_{y}} \end{array} \end{array}\right]$$

Finally, the absolute values for horizontal distance, vertical distance and inclination angle are defined for each frame according to the following expressions:

(8) $$d_{h,i}=\frac{1}{2}\cdot \left(L1,i^{\left(1\right)}{}_{x}+L2,i^{\left(1\right)}{}_{x}\right)\cdot \frac{d_{x}}{P2,i^{\left(1\right)}{}_{x}}$$

(9) $$d_{v,i}=\frac{1}{2}\cdot \left(L1,i^{\left(1\right)}{}_{y}+L2,i^{\left(1\right)}{}_{y}\right)\cdot \frac{d_{y}}{P3,i^{\left(1\right)}{}_{y}}$$

(10) $$tan\left(\theta_{i}\right)=\frac{L1,i^{\left(3\right)}{}_{y}-L2,i^{\left(3\right)}{}_{y}}{L1,i^{\left(3\right)}{}_{x}-L2,i^{\left(3\right)}{}_{x}}\cdot \frac{d_{y}}{P3,i^{\left(1\right)}{}_{y}}\cdot \frac{P2,i^{\left(1\right)}{}_{x}}{d_{x}}$$

In this case, “absolute values” refers to the values relative to the beginning of the measurement.

**Figure 6:**
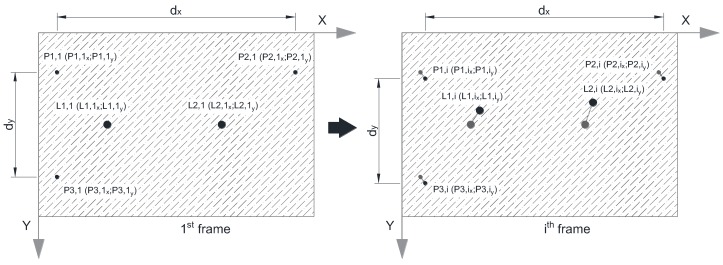
Relative movement of the five references between the first and the *i*th frame.

Between the 1st and the *i-*th frame there is, in a general case, a movement of all references, both the fixed and movable ones. In fact, a movement of the movable references *P*1,*i*, *P*2,*i*, and *P*3,*i* are only observed if, during the record, a relative movement between the video camera and the measurement panel occurs. However, even in this case and since the references *P*1,*i*, *P*2,*i*, and *P*3,*i* define an intrinsic coordinate reference and *L*1,*i* and *L*2,*i* are, in fact, fixed references, it is possible to define the real movement of the target, through the variation of the coordinates of *L*1,*i* and *L*2,*i* referred to the local axis *X*’-*Y*’, according to the following expressions and illustrated by Figure 6:

(11) $$\delta_{h}=d_{x,i}-d_{x,1}$$

(12) $$\delta_{v}=d_{v,i}-d_{v,1}$$

One of the main advantages of this proposed approach, and also its main novelty, is that every frame includes both the fixed and the movable references. In consequence, the measurement is independent of the position of the video camera. The system is capable of capturing both the horizontal and vertical movement as well as the rotation of the target, all of them contained in the measurement panel plane. The range of the measurement depends on the dimensions of the measurement panel and the resolution depends on the quality of the video camera. Another relevant advantage is that the post-processing is simple and computationally inexpensive, and can thus be performed on a regular desktop or laptop computer. The procedure supports every recording speed (from ultrahigh speed to time lapse recording) and every video camera resolution (from 720 p to the new 8K).

This solution provides sub-pixel accuracy for displacements, both horizontal and vertical. For the case of rotations, it is less accurate, since it is inversely proportional to the distance between laser dots.

It is not necessary that the laser beams should impact perpendicular to the measurement panel as long as the panel moves only within its plane. However, if out-of-plane movements and/or rotations of the panel are expected to occur (e.g., due to creep, shrinkage and thermal variations in case of concrete bridges and long-term monitoring, or even abnormal or non-expected movements and/or rotations), the lack of orthogonality between laser beams and measurement panel introduce non-real movements. These are proportional to cos(*θ*), where *θ* is the angle between the laser beams and the measurement panel. Consequently, it is recommended that the laser beams impact perpendicular to the measurement panel.

While the proposed solution has been primarily designed to measure static and low-varying displacements and rotations, the authors have plans to evaluate it for vibration measurements in the near future. Anticipated adjustments are related to frame rate and fixation of the movable part to ensure proper coupling.

## 3. Experimental Evaluation

### 3.1. Laboratory Testing

**Figure 7:**
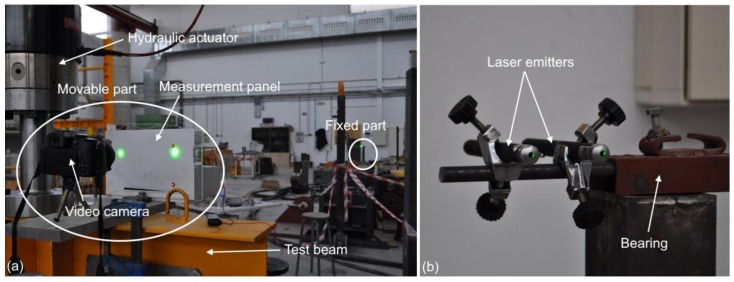
Laboratory setup: (**a**) General overview with movable part and (**b**) Close-up view of fixed part.

A laboratory experiment was performed to compare the proposed LVBDT with a traditional linear variable differential transformer (LVDT), which served as the reference measurement. The movable part of the LVBDT was placed on a horizontal steel beam connected to a dynamic tension—compression actuator, model MTS 244.51 (MTS, Eden Prairie, MN, USA), with a capacity of ±1000 kN. The actuator had a built-in LVDT with a range of 250 mm and an error < 1% over its range. The actuator was connected to an ultra-rigid frame. The fixed part of the proposed LVBDT was placed on the floor of the lab, at a distance of 15 m (Figure 7). The distance between camera lens and panel was 300 mm and the pixel size was approximately 0.08 mm.

**Figure 8:**
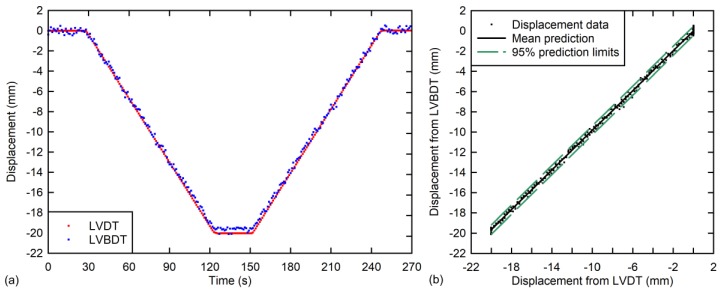
Results from laboratory experiment: (**a**) Measured vertical displacements vs. time for laboratory experiment and (**b**) LVBDT vs. LVDT correlation plot with 95% prediction limits.

During the test, the piston of the hydraulic actuator was moved down slowly, remaining at this position for several seconds and, finally, it was moved down up to its initial position. The maximum displacement was 20 mm. The displacement rate was 0.2 mm/s. During testing, the vertical deflection was measured and recorded using both the LVDT of the hydraulic jack and the proposed LVBDT. In this case, although the recording speed of the video camera was 60 fps, only 1 fps was used for post-processing. A comparison of the obtained displacements from both sensors are shown in Figure 8a.

As can be observed in Figure 8a, the displacement curves are in close agreement, i.e., the LVBDT shows very similar results compared to the reference measurement (LVDT). The maximum displacement difference was found to be 0.6 mm, which appears to be due to small variations in the color intensity of the pixels belonging to the different reference points or the presence of shadows that partially cut the reference point, thereby slightly affecting the center of gravity. The correlation between the two measurements is high, as can be observed visually in Figure 8b. A simple linear regression produced a slope of 0.98, that is, on average, the LVBDT gives displacements that are 2% lower compared to the LVDT displacements. The squared correlation coefficient was found to be 99.9%. The mean and standard deviation of the residuals, which are the differences between the displacements obtained from the two sensors, was 0.19 mm and 0.23 mm, respectively. In this test, no rotation was applied.

### 3.2. In-Service Field Testing

**Figure 9:**
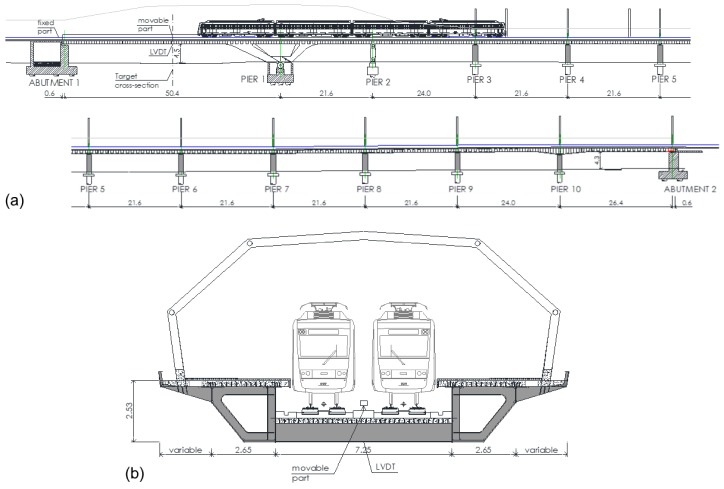
Loiola Station Bridge: (**a**) Elevation view with sensor locations and (**b**) cross-section at location of movable part (units in meters).

**Figure 10:**
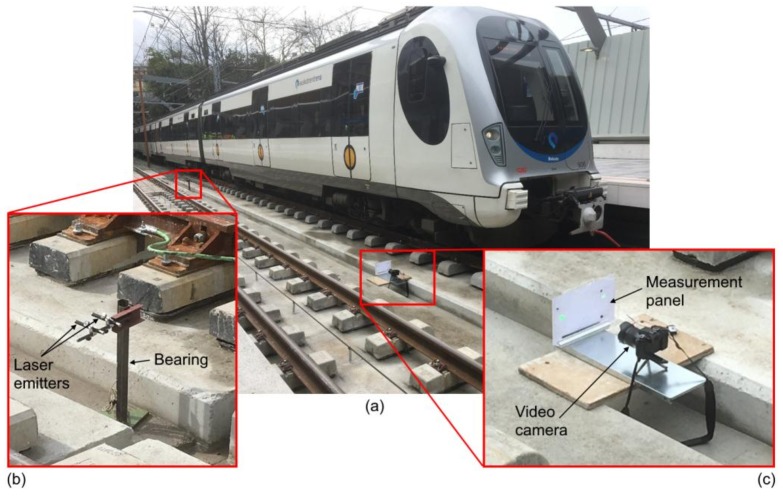
In-service field test setup: (**a**) General overview; (**b**) close-up view of fixed part; and (**c**) close-up view of movable part.

An in-service load test was performed in order to evaluate the response of the proposed LVBDT under realistic field conditions. The tested structure was the Loiola Station Bridge located in the City of San Sebastian, Spain and is an 11-span continuous composite steel-concrete bridge. The span lengths are 50.4 + 21.6 + 24.0 + 6 × 21.6 + 24.0 + 26.4 m corresponding to a total length 277.2 m. The cross section includes to steel box girders connected with transverse I-beam girders. Both the box and the I-beam girders have an in-situ concrete slab of 0.25 m thickness, as shown in Figure 9 and Figure 10.

During the load test, among other parameters, vertical deflection at the mid-span cross section of Span 1 was measured (Figure 9a). These displacements were measured using the proposed LVBDT as well as with a conventional LVDT. For case of the LVBDT, the fixed part was placed outside the deck, on the abutment between the two rail lanes, and the movable part was placed on the target cross-section, on the deck and between the two rail lanes. The distance between the fixed and the movable parts was approximately 26 m (Figure 9 and Figure 10). The distance between camera lens and panel was 300 mm and the pixel size was approximately 0.08 mm.

**Figure 11:**
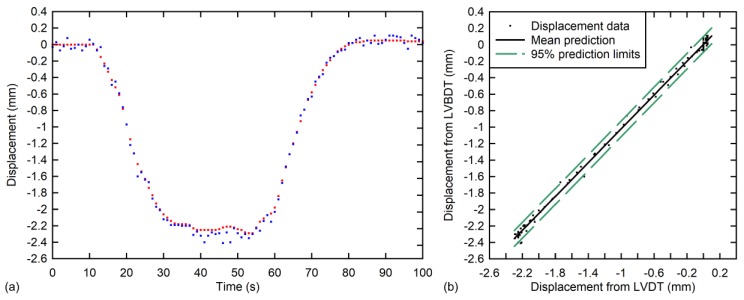
Results from laboratory experiment: (**a**) Measured vertical displacements vs. time for laboratory experiment and (**b**) LVBDT vs. LVDT correlation plot with 95% prediction limits.

During the load test, several trials were carried out by passing a train over the deck from Abutment 1 to Abutment 2 at different speeds (from low to high). Both LVDT and LVBDT recorded the variation of the vertical deflection of the mid-span cross section over time. Similarly to the lab experiment, although the recording speed of the video camera was 60 fps, only 1 fps was post-processed. A comparison of the obtained displacements from both sensors for one select trial are shown in Figure 11. In this case, the train speed was 5.0 km/h.

As can be observed in Figure 11a, the displacement curves are again in close agreement. The maximum displacement difference was found to be 0.2 mm. For the case of this field test, additional causes of discrepancies can be considered. The most important one being that the fixed part was placed on the abutment between the rail lanes, which may have been experiencing minute movements during testing, thereby affecting the measurement. The correlation of the two measurements is high, as can be observed visually in Figure 11b. A simple linear regression produced a slope of 1.026, that is, on average, the LVBDT gives displacements that are 2.6% higher compared to the LVDT displacements. The squared correlation coefficient was found to be 99.8%. The mean and standard deviation of the residuals, which are the differences between the displacements obtained from the two sensors, was 0.04 mm and 0.05 mm, respectively. Due to the very high torsional stiffness of this bridge, rotation could not be measured.

## 4. Summary and Conclusions

To date, long-term monitoring of vertical bridge deflections is a technological challenge not well solved. The different technical solutions that can be found on the market only partially address the requirements, especially for the case of large, tall and/or very stiff bridges, or for the ones located in remote places. Hence, an accurate, robust, reliable, and inexpensive solution is needed.

This paper presents a novel sensing approach to measure the displacements and rotations in bridges and structures using laser beams, LED lights, and a digital video camera, referred to as laser and video-based displacement transducer (LVBDT). This solution has been especially designed for the long-term monitoring of slowly varying displacements acquired during any time of the day. The LVBDT overcomes some of the limitations shown by the previous solutions. The main novelty is that, inside the field of vision, there are both the fixed and the movable references. In consequence, it is not necessary that the video camera remains fixed or moves jointly with the target; in fact, the position and/or the movement of the video camera during the monitoring has no impact on the measurement. Another advantage is the complete insensitivity to changing lighting conditions, which can be a significant problem for other contemporary video-based approaches. Contrary to other video-based approaches, the camera is located on the bridge, hence allowing to capture displacements at one location.

This paper describes the proposed sensor and the computational procedure to obtain the displacements and rotations. The post-processing scheme is simple and computationally inexpensive, and can be performed with off-the-shelf desktop or laptop computers. The laboratory experiment and in-service field test demonstrate the promise of the proposed approach. The differences between the proposed LVBDT and LVDT was within ±2%, which is acceptable for bridge deflection measurements. Future work will look at improving the computational approach, developing an integrated prototype, and evaluating the solution for vibration measurements.

## 5. Patents

A Spanish patent (Patent No.: P201730410) has been submitted and is currently pending [39].
